# Supplementary material for: Chromoblastomycosis in an Endemic Area of Brazil: A Clinical-Epidemiological Analysis and a Worldwide Haplotype Network
Source: J Fungi (Basel). 2020 Oct 3;6(4):204. doi: 10.3390/jof6040204 (PMC7711792; doi:10.3390/jof6040204)
Supplement: Supplementary file 1 [file jof-06-00204-s001.pdf]

Table S1. Strains of *Fonsecaea* spp. involved in the ITS world haplotype network and their global distribution.

| Haplotype number | Number of strains | Strain number | Countries | Agent |
|---|---|---|---|---|
| Hap1 | 19 | MF179511, AY857512, EU938600, EU938581, , KX434639, KX434635, KX434636, KX434637, KX434638, KX434641, EU938583, EU938579, AY366928, MF179511AY366926, EU938580, KP132195, KC283184, KC614700 | Brazil, China, USA, Cuba, Poland | *F. monophora* |
| Hap2 | 1 | KX434631 | Brazil | *F. monophora* |
| Hap3 | 1 | KX434632 | Brazil | *F. monophora* |
| Hap4 | 1 | KX434640 | Brazil | *F. monophora* |
| Hap5 | 3 | EU938582, EU938548, KP132194 | Brazil | *F. monophora* |
| Hap6 | 5 | FJ785471, KP132193, KY303869, KM225277, KY310638 | India, Guinea | *F. monophora* |
| Hap7 | 1 | EU938584 | USA | *F. monophora* |
| Hap8 | 1 | EU285268 | China | *F. monophora* |
| Hap9 | 2 | EU285270, EU285273 | China | *F. monophora* |
| Hap10 | 1 | MH382090 | Brazil | *F. monophora* |
| Hap11 | 1 | MH382084 | Brazil | *F. monophora* |
| Hap12 | 1 | KX434639 | Brazil | *F. monophora* |
| Hap13 | 28 | KC886421, KP132197, JQ906791, JN418760, JQ906793, JN418762, FJ595996, EF513771, EF513770, EF513769, EF513766, EF513765, EF513764, EF513761, KX078402, KX078386, EU285269, KX078401, KX078389, KX078388, EU285272, KX078380, KX078398, KX078399, KX078394, KX078387, KX078395, KX078392 | China | *F. monophora* |
| Hap14 | 1 | EF513768 | China | *F. monophora* |
| Hap15 | 1 | KM396287 | Malaysia | *F. monophora* |

| | | | | |
|---|---|---|---|---|
| Hap16 | 14 | KX078391, KX078397, KX078393, KX078382, EF513767, EF513760, EF513762, EF513763, EF513759, KX078400, KX078378, KX078390, KX078385, JN629041 | China | *F. monophora* |
| Hap17 | 2 | AY366926, AY366986 | Brazil | *F. monophora* |
| Hap18 | 1 | EU938579 | Brazil | *F. monophora* |
| Hap19 | 1 | AY366928 | Brazil | *F. monophora* |
| Hap20 | 1 | KC886423 | Brazil | *F. pedrosoi* |
| Hap21 | 2 | KX434682, KU881745 | Brazil | *F. pedrosoi* |
| Hap22 | 70 | KX434642, KR732301, KR732303, KR732304, KR732305, KX434643, KX434644, KX434645, KX434646, KX434647, KX434648, KX434649, KX434650, KX434651, KX434652, KX434653, KX434654, KX434655, KX434656, KX434657, KX434658, KX434659, KX434660, KX434661, KX434662, KX434663, KX434664, KX434665, KX434666, KX434667, KX434668, KX434669, KX434670, KX434671, KX434672, KX434673, KX434674, KX434675, KX434676, KX434677, KX434678, KX434679, KX434704, KX434680, KX434681, KX434682, KX434683, KX434684, KX434685, KX434686, KX434687, KX434688, KX434689, KX434690, KX434691, KX434692, KU881736, KU881744, KX434710, KX434717, KX434711, KX434712, KX434713, KX434714, AY366918, AY366920, AY366913, EU938588 EU938586, EU938587 | Brazil, Argentina, Lybia, Venezuela | *F. pedrosoi* |
| Hap23 | 7 | KX434693, KX434694, KX434695, KX434696, KX434697, KX434698, KX434699 | Brazil | *F. pedrosoi* |

| | | | | |
|---|---|---|---|---|
| Hap24 | 1 | KX434700 | Brazil | *F. pedrosoi* |
| Hap25 | 1 | KX434709 | Brazil | *F. pedrosoi* |
| Hap26 | 1 | AY366919 | Brazil | *F. pedrosoi* |
| Hap27 | 1 | KX434702 | Brazil | *F. pedrosoi* |
| Hap28 | 1 | KX434704 | Brazil | *F. pedrosoi* |
| Hap29 | 1 | KX434705 | Brazil | *F. pedrosoi* |
| Hap30 | 1 | KX434720 | Brazil | *F. pedrosoi* |
| Hap31 | 1 | KX434721 | Brazil | *F. pedrosoi* |
| Hap32 | 1 | KX434678 | Brazil | *F. pedrosoi* |
| Hap33 | 1 | AY366912 | - | *F. pedrosoi* |
| Hap34 | 1 | AY366915 | Puerto Rico | *F. pedrosoi* |
| Hap35 | 1 | AY366921 | Uruguay | *F. pedrosoi* |
| Hap36 | 1 | KX434701 | Brazil | *F. pedrosoi* |
| Hap37 | 1 | KX434703 | Brazil | *F. pedrosoi* |
| Hap38 | 1 | KC886422 | Brazil | F. pedrosoi |
| Hap39 | 2 | KU892412, KU881735 | Brazil | *F. nubica* |
| Hap40 | 1 | KX084063 | China | *F. nubica* |
| Hap41 | | KF155217, KP132198, MF038001, KX084064, KX078407, KX078410, KX078404 | China | *F. nubica* |
| Hap42 | 1 | KX078408 | China | *F. nubica* |
| Hap43 | 1 | KU885982 | China | *F. nubica* |
| Hap44 | 1 | KX084065 | China | *F. nubica* |
| Hap45 | 1 | AY366931 | Suriname | *F. nubica* |
| Hap46 | 1 | EU938592 | Camaroon | *F. nubica* |
| Hap47 | 3 | KP132199, KX084066, KX078409 | China | *F. nubica* |
| Hap48 | 1 | KX078403 | China | *F. nubica* |
| Hap49 | 1 | KX078406 | China | *F. nubica* |
| Hap50 | 1 | KX078405 | China | *F. nubica* |
| Hap51 | 1 | EU938589 | Mexico | *F. pedrosoi* |

| | | | | |
|---|---|---|---|---|
| Hap52 | 1 | MH858792 | Venezuela | *F. pedrosoi* |
| Hap53 | 1 | KU881739 | Brazil | *F. nubica* |
| Hap54 | 1 | EU938591 | Puerto Rico | *F. pedrosoi* |
| Hap55 | 1 | EU938590 | Mexico | *F. pedrosoi* |
| Hap56 | 1 | KU892413 | Brazil | *F. pedrosoi* |
| Hap57 | 1 | KU8811742 | Brazil | *F. nubica* |
| Hap58 | 1 | KR706553 | Brazil | *F. pugnacius* |
| Hap59 | 1 | EU137319 | Brazil | *F. monophora* |
